# A new scaffold-free tumoroid model provides a robust preclinical tool to investigate invasion and drug response in Renal Cell Carcinoma

**DOI:** 10.1038/s41419-023-06133-z

**Published:** 2023-09-22

**Authors:** Irinka Séraudie, Catherine Pillet, Beatrice Cesana, Pauline Bazelle, Florian Jeanneret, Bertrand Evrard, Frédéric Chalmel, Assilah Bouzit, Christophe Battail, Jean-Alexandre Long, Jean Luc Descotes, Claude Cochet, Odile Filhol

**Affiliations:** 1 University Grenoble Alpes, Inserm, CEA, IRIG-Biosanté, UMR 1292, F-38000 Grenoble, France; 2 University Rennes, Inserm, EHESP, Irset (Institut de Recherche en Santé, Environnement et Travail) - UMR_S 1085, F-35000 Rennes, France; 3 Centre hospitalier universitaire Grenoble Alpes, CS 10217, 38043 Grenoble, cedex 9, France; 4 Present Address: University Grenoble Alpes, Inserm, CEA, IRIG-Biosanté, UA 13, F-38000 Grenoble, France

**Keywords:** Renal cell carcinoma, Experimental models of disease

## Abstract

Clear cell Renal Cell Carcinoma (ccRCC) is one of the most prevalent kidney cancers, which is often asymptomatic and thus discovered at a metastatic state (mRCC). mRCC are highly heterogeneous tumors composed of subclonal populations that lead to poor treatment response rate. Several recent works explored the potential of ccRCC tumoroids culture derived from patients. However, these models were produced following a scaffold-based method using collagen I or Matrigel that exhibit lot variability and whose complexity could induce treatment response modifications and phenotypic alterations. Following the observation that ccRCC tumoroids can create their own niche by secreting extracellular matrix components, we developed the first scaffold-free tumoroid model of ccRCC tumors. Tumoroids from mice as well as from human tumors were generated with high success rate (≥90%) using a magnetic suspension method and standard culture media. Immunofluorescence analysis revealed their self-organization capacities to maintain multiple tumor-resident cell types, including endothelial progenitor cells. Transcriptomic analysis showed the reproducibility of the method highlighting that the majority of gene expression patterns was conserved in tumoroids compared to their matching tumor tissue. Moreover, this model enables to evaluate drug effects and invasiveness of renal cancer cells in a 3D context, providing a robust preclinical tool for drug screening and biomarker assessment in line with alternative ex vivo methods like tumor tissue slice culture or in vivo xenograft models.

## Introduction

Kidney cancer represents the 8th most frequent type of cancers in the word [1]. Among them, Renal Cell Carcinoma (RCC) is the most common and englobes a number of histological subtypes comprising clear cell RCC (ccRCC) and papillary RCC. The most frequent is ccRCC, which represents 70–80% of patients, followed by papillary RCC, which represents 10–15% [2, 3]. RCC patients are often asymptomatic, leading to 30% of metastatic RCC (mRCC) at diagnosis. Moreover, 10% of patients that are undergoing surgery for a localized cancer will relapse and develop mRCC during their follow up [4]. mRCC can be treated with Tyrosine Kinase Inhibitors (TKI) or Immunotherapy [5]. Recently, a combination of Immune checkpoint inhibitors like Ipilimumab plus Nivolumab has been approved for Metastatic Renal Cell Carcinoma management [6]. However, intrinsic and therapy-induced heterogeneity, and changes in the tumor microenvironment often yield to multiple cross-resistance mechanisms in non-responder patients. Phylogenic studies showed that subclonal populations with different mutations arise from the trunk mutations that are present in all regions of the tumor before formation of the founder cancer cells. Those subclones are often responsible of metastasis and are not many to be detected by bulk sequencing [6–9]. This leads to mRCC patients with a therapeutic response that differs significantly independently of their trunk mutation status [10, 11].

Tumors appear as complex systems with many elements that display dynamic spatial and temporal evolution as they progress and respond to stressors, including the immune system and cancer therapies. Recently, advances in tumor models have demonstrated the value of organoid technology for the generation of in vitro three-dimensional models, including patient-derived organoids that have been shown to more accurately recapitulate the structures, specific functions, molecular characteristics, genomic alterations and tumor microenvironment of primary tumors. In addition, they may be useful for identifying in vitro drug response as well as biomarker assessment to help guiding personalized therapy and molecular profiles to define predicative molecular profiles of drug responsive tumors [12–14]. The need for such a dynamic system is emphasized by the high failure rate of drugs tested in clinical trials. Indeed, the success rate for cancer treatments is 3.4% in phase I–III clinical trials [15]. Sato et al. developed in 2011, the first tight irregular cancer cell aggregates that appeared similar to in vivo tumors, nowadays referred to as tumoroids [16]. Subsequently, tumoroids of numerous cancer types were established [14], including prostate [17], ovarian [18], brain [19, 20], bladder [21], gastric [22–24], esophageal [25] revealing that patient-derived tumoroids recapitulate patient-specific histological features. However, their use in the field of kidney cancers remains limited [26–30]. Over all those methods, establishment success rates varied widely, across the different sources of tumor tissue they were generated from. They can be derived from primary tumors, metastatic lesions, tumor cells from liquid effusion or circulating tumor cells [13]. Following collection, processing of tumor samples can be achieved with different strategies like enzymatic and/or mechanic dissociation, and tumoroids can be formed from progenitor single cell, multicellular reassembly or millimeter-scale tumor fragments [17, 22]. Several recent works describe the establishment and characterization of ccRCC organoid cultures derived from patients’ tissues revealing biological insights for ccRCC research [25, 27–30]. However, for their establishment, these organoid models rely on their plating on low-density Matrigel® or type 1 Collagen matrices that might introduce unknown variables into culturing experiments. Thus, there is an emerging need to develop Matrigel®-independent organoid culture methods. Despite the assumption that a matrix is required to sustain long-term organoid culture, it was hypothesized that cells in the organoid may create their own niche by secreting extracellular matrix components. This led to the development of matrix-free culture systems for a narrow range of tissues [31–33]. In the present work, we describe the establishment, characterization and several applications of a matrix-free technique for the culture of renal tumor organoids (tumoroids) derived from renal tumor xenografts in mice or human tumor surgical specimen. We provide a phenotypical, transcriptomic and molecular analysis of these organoids and the corresponding tumors. Moreover, we found a clear concordance of the tumor response to treatments in this tumoroid culture model compared to tumor slice cultures or orthotopic renal cancer xenografts.

## Results

### Establishment of ccRCC tumoroid cultures

**Fig. 1:**
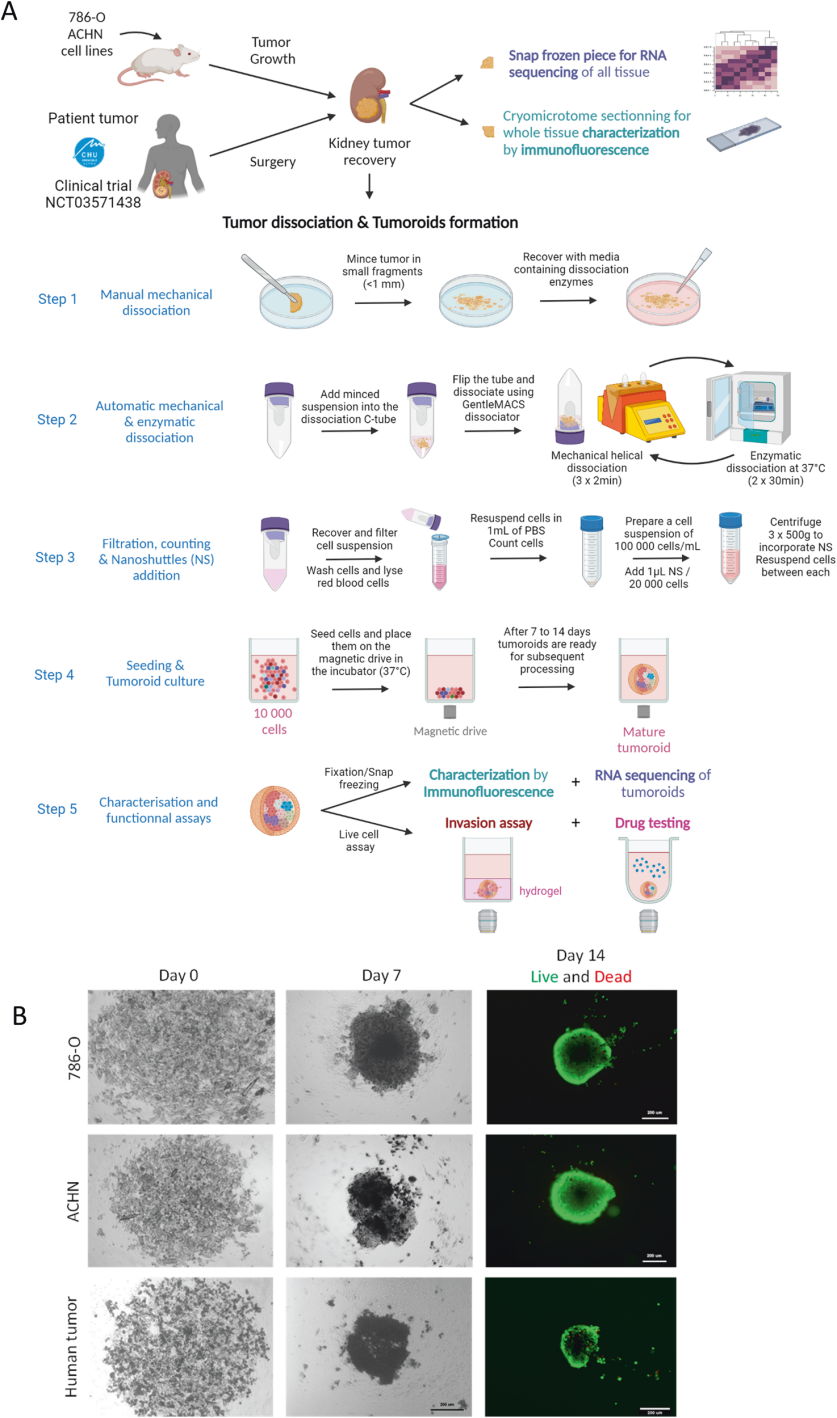
Generation of tumoroids from mice tumors and human tumors. **A** 786-O-Luc and ACHN-Luc cells are surgically implanted under renal capsula (1. 10^6^ cells/mice) and tumor growth is followed using luminescence (IVIS imager). Mice are euthanized when tumor growth is above 1. 10^9^ photon/sec/cm². Human tumors are recovered fresh just after surgery at Grenoble Alpes Hospital. Kidney tumors are split into 3 pieces: 2 are fresh frozen for RNA sequencing and cryomicrotome sectioning. The third one is used to produce tumoroids in culture. Tumors are mechanically and enzymatically dissociated. One fraction is fresh frozen while the second fraction is coated with iron nanoparticles to produce tumoroids thanks to a magnetic spheroid drive. After 1 or 2 weeks of maturation, tumoroids are characterized by immunofluorescence and RNA sequencing and they are used for invasion assay and drug testing. **B** Example of tumoroids’ maturation from ACHN, 786-O and ccRCC human tumors during 7 days. At 14 days, viability assay (Live&Dead, using Calcein, Hoechst and Ethidium homodimer) is performed on tumoroids. Scale bar = 200 µm.

We generated renal tumor in mice by surgically implanting 786-O-Luc or ACHN-Luc cells under the renal capsula. 786-O cell line is a classically used ccRCC cell line that harbors VHL mutation and constitutively active HIFα, while ACHN cell line is defined as papillary RCC without VHL mutation [34, 35]. Human ccRCC specimen were collected fresh at the time of surgical resection at the Grenoble hospital and taken immediately to the laboratory for processing (Clinical trial NCT03571438). Several pieces of each tumor were snap frozen for RNA isolation and cryo-microtome sectioning. The remainder was mechanically and enzymatically dissociated. After counting, dissociated cells were seeded to perform tumoroid culture, using a scaffold-free tumoroid method (Fig. 1A). After 7 days, formation of live tumoroids was clearly visible by microscope evaluation. Tumoroids were viable for at least 14 days (Fig. 1B). Thus, with a success rate of 100% in mice tumor samples and 90% in human tumors, this model allows an accurate comparison to evaluate its translational potential with other effective pre-clinical cancer models.

### Phenotypic characterization of ccRCC tumoroids

**Fig. 2:**
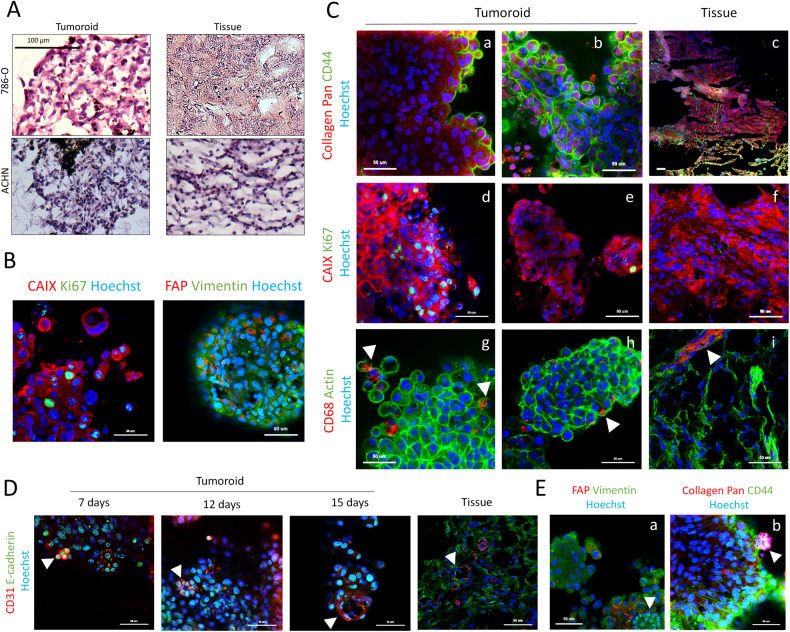
Phenotypic characterization of mice tumoroids by immunohistochemistry and immunofluorescence. **A** 786-O and ACHN tumoroids and their counterpart tissue stained with Hematoxylin and Eosin (HE). Images were taken with AxioVision Zeiss microscope. Scale bar = 100 µm. **B**–**E** Immunofluorescence images of ACHN tumoroids and tissue that were taken using the LSM 880 AiryScan confocal microscope (Zeiss). Scale bar = 50 µm. All samples are stained with Hoechst. **B** Tumoroids labeled with CAIX, Ki67, Vimentin and FAP. **C** Tumoroids and their corresponding tissue labeled with Collagen Pan and CD44 (a–c), CAIX and Ki67 (d–f) and CD68 and actin (g–i). **D** Tumoroids labeled with CD31 and E-cadherin at different time point (7, 12 and 15 days) and the corresponding tissue. **E** Tumoroids labeled with FAP and vimentin (a), and Collagen Pan and CD44 (b).

To determine whether phenotypic features are preserved in vitro, we histologically characterized the ccRCC tumoroids. Gross morphology of tumoroids as well as examination of hematoxylin and eosin (H&E) staining revealed that they generally resembled the parental tumor tissue. Like in matching tumor tissue, 786-O-derived tumoroids consist of dense packed cells with a filled cytoplasm, an histological feature already reported in mouse xenograft models [9], while ACHN show tubulo-papillary architecture with clear cell-like areas (Fig. 2A). In most tumoroids, different cell clusters could be distinguished demonstrating a rather heterogeneous composition of different sub-populations (Supp Fig. 1). The major one is represented by cancer cells, assessed by the expression of vimentin and the classical ccRCC-specific marker CAIX [3, 36]. A second population showed strong enrichment for stromal markers such as FAP, thus likely representing fibroblasts (Fig. 2B). Interestingly, Pan Collagen labeling was mainly intracellular, suggesting that cells were able to produce de novo collagen to build their own extracellular matrix (Fig. 2C a, b) [37]. Enrichment for the progenitor-like marker CD44 was observed mostly at the periphery of several tumoroids (Fig. 2C a, b), as in the parental tumor tissue (Fig. 2C c). While Ki67 staining was weak in tumor tissues (Fig. 2C f), this proliferation index was restored in growing tumoroids (Fig. 2C d,e). Clusters of CD68^+^ cells were observed at the periphery of the tumoroids (Fig. 2C g, h), likely representing tumor-associated macrophages (TAMs) that are the most abundant immune cell population infiltrating the tumor microenvironment (Fig. 2C i) [38, 39]. Interestingly, at day 12 and 15 of culture, both 786-O and ACHN tumoroids disclosed CD31^+^ cells that form circular layers of oriented packed cells at the surface of the structure, tentatively evoking a primitive vascular-like plexus as in the original tissue (Fig. 2D and S1). These CD31^+^ cells also expressed vimentin (Fig. 2E a, white arrow) and more importantly collagen, in higher proportions than other cell types (Fig. 2E b, white arrow), suggesting that CD31^+^ cells may start to secrete collagen IV that has been shown to be necessary to create their basement membrane [40, 41].

**Fig. 3:**
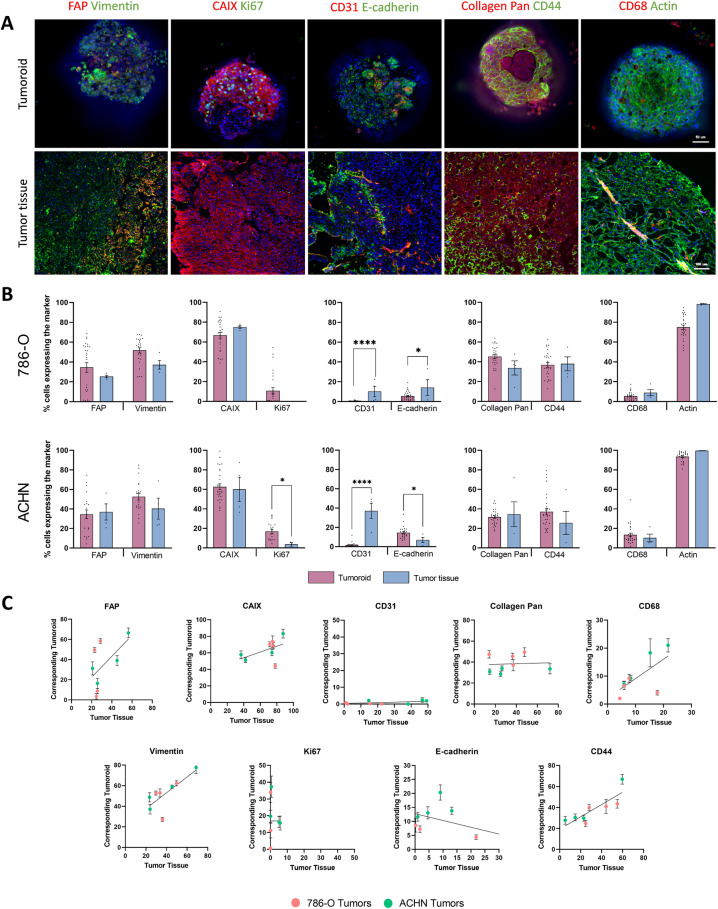
Cell populations proportions retained in 786-O and ACHN tumoroids compared to their original tumor tissue. **A** Immunofluorescent images of 786-O whole mounted tumoroids and their counterpart tissues. Tissue sections are 8 µm thick. Each column represent a different co-labeling : (1) FAP & vimentin, (2) CAIX & Ki67, (3) CD31 & E-cadherin, (4) collagen Pan & CD44, (5) CD68 & actin. Images were obtained using the Cell In Sight CX7 High throughput automated microscope (ThermoFisher) using 20x objective (NA 0.45) for tumoroids and 10x objective (NA 0.4) for tissue slices. Tumoroid scale bar = 50 µm; Tissue scale bar = 100 µm. **B** Percentage of cells expressing the different markers in tumoroids and tissues in a grouped analysis. 786-O tumoroids and ACHN tumoroids were respectively grouped to obtain global proportions. Quantification of different populations was made by the Thermo Fisher’s HCS studio software using the spot detector bioapplication. Reference levels for positive cells to each marker was the same for all samples. Quantification was made on the following number of replicates : Tumoroids 786-O = 27; Tumoroids ACHN = 20–28; Tissues = 4. Statistical analysis was made using student *t*-test, *p* < *0.05, ****0.0001. **C** Correlation and linear regression of the percentage of cells that are positive for a markers in tumoroids compared to their respective tissue they are derived from. *X*-axis correspond to the percentage of positive cells for a marker present in the tissue while Y-axis correspond to the mean of the percentage of positive cells for 6 to 8 tumoroids per tumor. Correlations are significant for FAP, vimentin, CAIX, CD68 and CD44 (*p* < 0.05).

Figure 3A shows immunofluorescence images of 786-O tumoroids and the corresponding parental tumors stained with antibodies directed against different cell-specific biomarkers. Figure 3B illustrates the grouped analysis of their expression levels in 786-O or ACHN tumoroids compared to the original tumor tissue (4 tumoroids grouped per cell line). As previously shown in Fig. 2, CD31^+^ cells were detectable in the tumoroids albeit with a reduced expression compared to the parental tumors, suggesting that angiogenesis is weak in growing tumoroids. Of note, many cell nuclei in these tumoroids, were positive for Ki67 staining as compared to the original tissue, indicating that actively dividing cells were still present after >2 weeks of culture. Surprisingly, compared to original tumor tissues, E-cadherin expression was lower in the 786-O tumoroids while higher in the ACHN tumoroids. In contrast, both types of tumoroids showed a similar expression of CAIX, vimentin, FAP, CD44 and CD68 as compared to parental tissues. In summary, this analysis reveals that different tumor-resident cell types such as cancer cells, fibroblasts, CD44 progenitor-like cells and infiltrated macrophages were preserved in these tumoroids.

We then performed a pairwise correlation of expression of cell-specific markers in tumoroids and their respective tumor tissue (Fig. 3C). As previously described, no clear correlation was found for CD31 and Ki67 expression. Compared to the original tumor tissues, CD31^+^ cells were reduced in the tumoroids while cell proliferation was increased. In contrast, significant correlations were found for FAP, vimentin, CAIX, CD68 and CD44, thus indicating that tumoroids are able to retain the inter-tumor heterogeneity present in the tumor tissues. Finally, ~40% of the cells in the tumoroids were producing a collagen matrix, regardless of its amount present in their respective original tissue. This suggests that tumoroids are capable to synthetize de novo collagen, in order to restore the ECM lost during the enzymatic dissociation process.

Altogether, these data indicate that these renal tumoroids display diverse differentiated cell types that are present in their respective original tumor tissue. Moreover, the tumoroids preserve the integrated stroma, which includes the extracellular matrix, vascular-like structures and other cell types.

### Gene expression of tumoroids and tumor tissues

To evaluate genetic similarities between tumoroids and the corresponding tumor tissues, we performed RNA sequencing of cells for each experimental condition (see “Methods”). Since we injected human cancer cells into mice, cancer cells in the tumors were human while microenvironment cells (i.e. Fibroblasts, Immune cells,…) were derived from mice. Thus, the RNA sequencing datasets generated from ACHN or 786-O tumor tissues (AxM and 7xM) and their respective tumoroids (AxTy and 7xTy) were mapped on human and mouse reference genome.

**Fig. 4:**
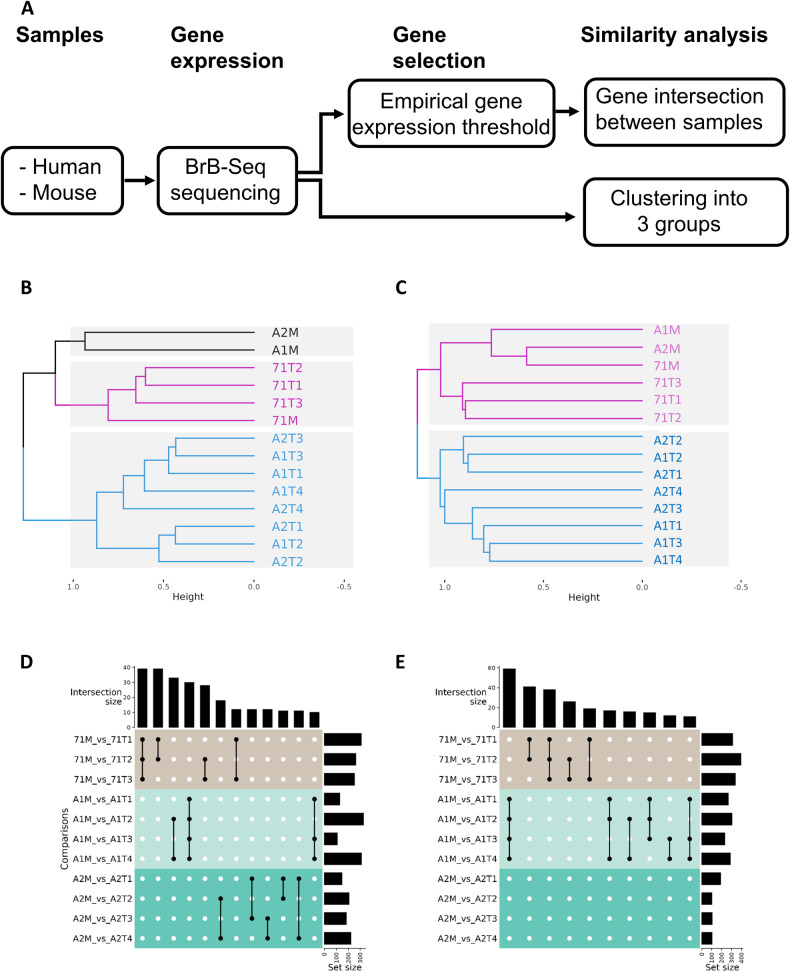
mRNA expression analysis of tumoroids versus tumor tissue. **A** Working pipeline. **B**–**E** 7xM and AxM are tumoral tissue samples while AxTx and 7xTx are their corresponding tumoroids. For example, 786-O tumor number 1 is named 71 M and its corresponding tumoroids are 71T1, 71T2 and 71T3; ACHN tumor number 1 is named A1M and its corresponding tumoroids are A1T1, A1T2, A1T3 and A1T4. **B**, **C** Dendogram representing the hierarchical clustering analysis for the mapped datasets in the human genome (**B**) and mouse genome (**C**) with 3 and 2 clusters respectively. **D**, **E** Intersection plots of comparisons between tissues and matching tumoroids for human (**D**) and mouse (**E**) mapped datasets. Set size is the number of selected genes based on a threshold applied on the log2 (fold change) from gene expression values comparisons between the M and T samples in M_vs_T labels. Intersection size were plotted only if ≥10.

We initially performed unsupervised hierarchical clustering analysis on gene expression values for human cancer cells and mice microenvironment cells (Fig. 4A, B respectively). For cancer cells (Fig. 4A), all ACHN tumoroids (AxTy) were grouped as their tumoral tissues (AxM). Moreover, 786-O tumoroids matched with tissue 71 M, indicating that tumoroids and the tumoral tissue shared a high similarity in cancer cell transcriptional profiles. Looking at the microenvironmental cells (Fig. 4B), all tumor tissues were clustered together, suggesting that the in vivo tumor microenvironment is quite stable, independently of the cancer cell line. Moreover, ACHN and 786-O tumoroids were respectively forming a cluster. This means that the microenvironment of all tumoroids derived from the same cancer cell line were close, advocating for a good reproducibility of the tumoroid production protocol. More precisely, the method kept the same proportion and cell diversity in all tumoroids produced from the same tumor. 786-O tumoroids were closer to their original tumor tissue compared to ACHN tumoroids in agreement with the observation that 786-O tumors are often more homogeneous than ACHN tumors in vivo. Of note, ACHN tumor tissues and tumoroids were not grouped together, indicating that tumoroids have lost some of the microenvironment cells, which may correspond to endothelial cells as described before (Fig. 3B, C). Also, branching lengths separating the cluster of samples were smaller for human cancer cells (Fig. 4A) compared to microenvironment cells (Fig. 4B), suggesting a greater heterogeneity between tumoroids at the microenvironment level compared to tumor cells.

We then performed comparisons between tissues (AxM or 7xM) and their respective tumoroids (AxTx or 7xTx) for cancer and microenvironment gene expression data (Suppl. Table 1 and Table 2). For each sample pairwise comparison, we identified differentially expressed genes using a Log2 fold change plot. This consisted of counting the number of conserved genes for a range of thresholds of [Log2 fold change] and selecting the threshold corresponding to the inflection point of the curve. From ~12,000 expressed genes, we carried out pairwise differential gene expression analyzes between a tissue and its tumoroids. Between 100 and 400 genes were identified as differentially expressed highlighting high similarities between tumoroids and their corresponding tumor tissues, either for human cancer cells (Fig. 4C) or microenvironment mouse cells (Fig. 4D). Moreover, an intersection size analysis (black dots and lines Fig. 4C, D), considering sets of more than 10 genes, showed that many deregulated genes were shared between the tumoroids produced from the same tumor, whereas no common deregulation was observed between tumoroids derived from different tumors. Ontological enrichment analyzes were performed from these lists of differentially expressed genes. However, the low number of deregulated genes did not reveal pathways of interest for interpreting the differences between tissue and tumoroid (data not shown).

Altogether, these data reveal that most of the tumoroids can preserve gene expression features associated with both the main tumor-resident and microenvironment cells of the source material. Importantly, the rather limited variability in the number of deregulated genes observed in the tumoroids compared to the same tumor tissue could be due to cell reprogramming imposed by enzymatic deconstruction of the tumor tissue and cell suspension culture that may affect the original cell-cell and cell-ECM interactions. Finally, the higher variability of gene expression that we observed in the tumoroid microenvironment is reminiscent of the intra-tumor spatial genetic heterogeneity described in ccRCC [42].

### Three-dimensional tumoroid invasion

**Fig. 5:**
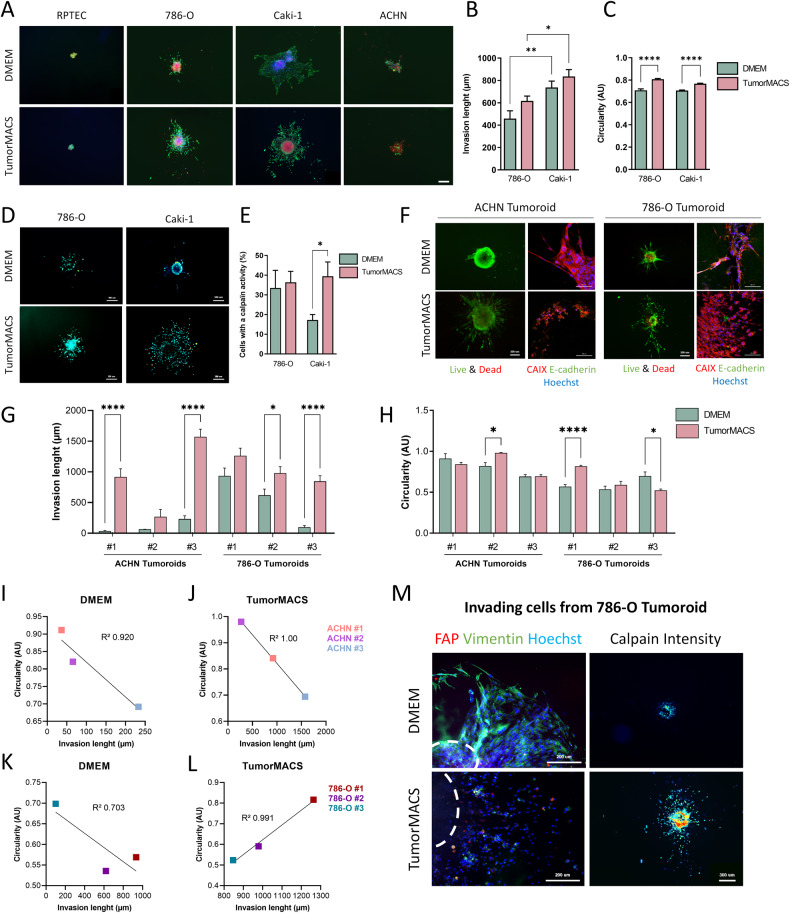
Invasion modes of spheroids and tumoroids in TissueSpec Kidney hydrogel. **A**–**E** Spheroids from cell lines that were embedded in TissueSpec Kidney hydrogel and incubated with DMEM supplemented or Tumor MACS (*n* = 10). RPTEC are healthy kidney cells, 786-O and Caki-1 are ccRCC cell lines and ACHN is a papillary RCC cell line. **A** Live & Dead staining (Calcein, Ethidium Homodimer) performed after 7 days of culture. **B** Invasion was quantified by measuring the invasion length from the border of the spheroid up to the farest cell. **C** Cell circularity of invading cells was quantified, 0 meaning straight line while 1 means circle. **D** Calpain-2 activity in spheroids using CMAC labeling and **E** quantification of invading cells with a calpain-2 activity after 7 days of culture. **F** ACHN and 786-O tumoroids derived from mice tumors that were embedded in TissueSpec Kidney hydrogel. After 14 days, tumoroids were labeled with Live and Dead staining (scale bar = 300 µm), and invading cells were fixed and stained for CAIX and E-cadherin (scale bar = 100 µm). **G** quantification of Invasion length or **H** cell circularity after 7 days of invasion (*n* = 2 tumoroids per tumor). **I**–**L** Pairwise correlation of invasion length (*x*-axis) and circularity (y-axis) for ACHN and 786-O tumoroids invading the hydrogel feeded with the indicated media. **I** ACHN tumoroids cultured in DMEM or **J** TumorMACS and **K** 786-O Tumoroids cultured in DMEM or **L** in TumorMACS. **M** Labeling of Mesenchymal cells (Vimentin), Fibroblasts (FAP) (scale bar = 200 µm) and Calpain-2 activity (CMAC) (scale bar = 300 µm) in cells that egress from 786-O tumoroids cultured in DMEM or TumorMACS after 14 days of culture. White dotted lines represent the emplacement of the tumoroids. Statistical analysis for histogram is student *t*-test while linear regression was performed on the correlation plots. *p < 0.05, **p < 0.01, ***p < 0.001, ****p < 0.0001.

Invasion is one of the first mechanisms that cancer cells use to escape from a primary tumor before metastatic colony formation in distant organs. To examine invasion in a 3D physiological context, spheroids generated from healthy kidney cells (RPTEC) or ccRCC cells (786-O, Caki-1) or papillary RCC cells (ACHN) were fully embedded within TissueSpec Kidney® hydrogel and incubated with different cell culture media. We then determined the cell capacity to escape from spheroids, to migrate and later on, to survive in the surrounding 3D matrix after 7 days of culture. As shown in Fig. 5A, no sign of invasion was detected in RPTEC spheroids. In contrast, cells from spheroids generated from the three different renal cancer cell lines, actively invaded at variable extents in this functional assay. Unexpectedly, in either DMEM or TumorMACS media, the invasive capacity of ACHN cells that are established as invasive, was weak as compared to Caki-1 and 786-O cells. Moreover, their invasiveness as well as their cell circularity were statistically increased in the TumorMACS® medium (Fig. 5B, C). Cancer cell invasion is known to be regulated by Calpain-2-mediated cleavage of the focal adhesion and invadopodia-associated protein talin [43, 44], leading to ameboid conversion of disseminated cells [45]. As depicted in Fig. 5D, E, Calpain-2 activity was clearly detectable after 7 days of culture and significantly increased in cells escaping from Caki-1 and 786-O spheroids cultured in TumorMACS® medium, suggesting an ameboid-like mode of invasion [46].

We next examined cell invasion using mice-derived tumoroids. Cells egressing from ACHN or 786-O tumoroids and invading the surrounding matrix were viable after 7 days (Fig. 5F). The escaping cells, which were identified as renal cancer cells disclosed a round-shaped phenotype in TumorMACS® compared to DMEM (CAIX/E-cadherin staining). Notably, cell egress from these tumoroids was strongly increased in the TumorMACS® medium (Fig. 5G), while cell circularity in both types of tumoroids was variable (Fig. 5H). Pairwise comparisons of invasion length and circularity show that in both culture media, cells escaping from ACHN or 786-O tumoroids with the lowest circularity, displayed enhanced invasiveness (Fig. 5I, J, K). In contrast, in the presence of TumorMACS® (Fig. 5L), invading cells from 786-O tumoroids had a different migration behavior with enhanced circularity leading to a more deformable and viscous phenotype [47]. This behavior can be possibly correlated with their high Calpain-2 activity and low vimentin expression (Fig. 5M).

Altogether, data from these studies highlight that both culturing conditions and the cell characteristics are important determinants affecting the migration mode of invading cells. In particular, the TumorMACS® medium may contain a signaling molecule composition that could promote round-shaped ameboid-like migration of 786-O cells, while DMEM does not [48, 49]. In line with this, 786-O cells that have a constitutive activation of HIFα, would be more receptive and prone to Calpain-2-mediated ameboid migration [45].

### Comparative responses to treatment on ex vivo and in vivo tumor models

**Fig. 6:**
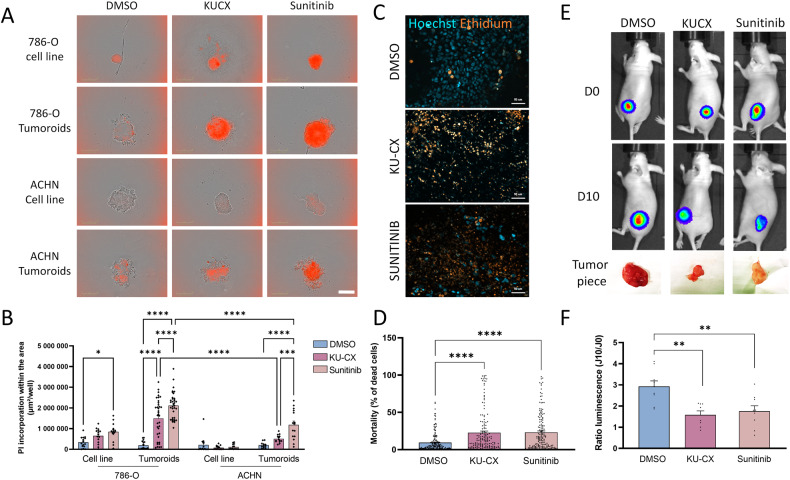
Assessment of tumoral response to treatment on different tumor models. **A**–**D** Spheroids, tumoroids and tissue slices were treated with KU-60019 (10 µM) plus CX-4945 (10 µM), Sunitinib (10 µM) or DMSO as control. **A**, **B** Spheroids and tumoroids were treated for 48 h and mortality was assessed with propidium iodide (0.5 µg/mL) using Incucyte zoom Imager, *n* = 12/18, scale bar = 300 µm. **C**, **D** Tumor slices were culture on air liquid interface and after 48 h they were labeled with Hoechst and Ethidium homodimer. **C** Pictures were taken using Zeiss Apotome microscope (20x, NA 0.7, scale bar = 50 µm) and analyzed with ImageJ and R studio to obtain percentage of dead cells (*n* = 13 mice) (**D**). **E**, **F** Three weeks old mice were subcutaneously injected with 8. 10^6^ 786-O Luc cells. After tumor growth they were treated for 10 consecutive days by force-feeding with KU-60019 (25 mg/kg) plus CX-4945 (12,5 mg/kg) or Sunitinib (50 mg/kg) or DMSO as control. **E** Luminescence follow up of tumors during treatment (days 0 and 10) and the piece of tumors after the sacrifice. **F** Ratio of luminescence of tumors before and after treatment (*n* = 8 mice per group). Kruskall–Wallis and Mann–Whitney statistical analysis were performed for in vitro and in vivo experiments respectively. *p < 0.05, **p < 0.01, ***p < 0.001,****p < 0.0001.

Various preclinical studies have been used as models to study responses to chemotherapies, although only a few have made direct clinical comparisons to responses in patients [50]. 786-O and ACHN cells grown as spheroids or as tumoroids derived from mice tumor samples were subjected to the FDA-approved compound sunitinib, used for first-line treatment of ccRCC or to KU-60019 plus CX-4945 (KU-CX), a combined inhibition of ATM and CK2 kinases, which has shown promising activities in a previously published drug screen [10]. After 48 h of treatment, spheroids as well as tumoroids were highly sensitive to sunitinib or the KU-CX combination (Fig. 6A, B). Similarly, renal tumor slice cultures displayed sensitivity to the same drug concentrations (Fig. 6C, D). Thus, three different models (spheroids, tumoroids, and tumor slices) derived from the same ccRCC sample showed similar early drug sensitivities. We also performed xenograft studies by subcutaneously injecting 786-O luc cells into mice. Oral drug administration started when average tumor size reached about 250 to 300 mm^3^. Mice were treated for ten consecutive days with the KU-CX combination or Sunitinib (Fig. 6E, F). In concordance with the effect observed on the tumoroid or the tumor slice models, growth inhibition of the corresponding tumor in mice matched the effective concentrations of sunitinib or the drug combination, validating our renal tumoroids as a relevant model for biological screening of anti-cancer compounds.

### Phenotypic characterization and treatment response of human renal tumoroids

**Fig. 7:**
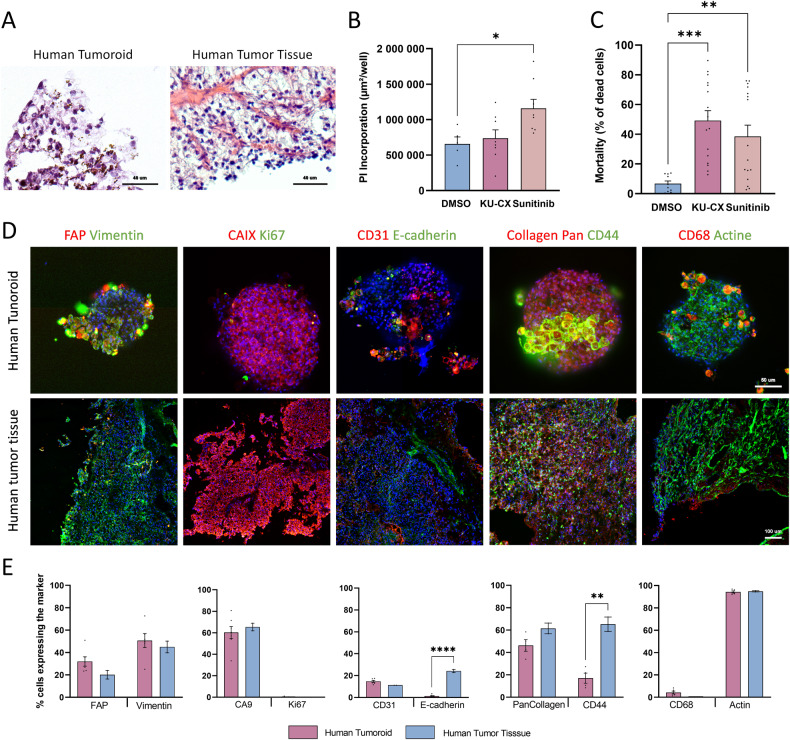
Characterization and drug sensitivity of the human ccRCC tumoroids. **A** Human tumoroid and its counterpart tumoral tissue from patient NJ115 stained with HE. Scale bar = 40 µm. **B**, **C** Tumoroids (**B**) and tissue slices (**C**) treated with KU-CX (10 µM each) or Sunitinib (10 µM) or DMSO as control. Mortality was assessed as in Fig. 5. **D** Immunofluorescent images of NJ115 whole mounted tumoroids and its counterpart tissue. Tissue sections are 8 µm thick. Each column represent a different co-labeling: (1) FAP & vimentin, (2) CAIX & Ki67, (3) CD31 & E-cadherin, (4) collagen Pan & CD44, (5) CD68 & actin. Images were obtained using the Cell In Sight CX7 High throughput automated microscope (ThermoFisher) using 20x objective (NA 0.45) for tumoroids and 10x objective (NA 0.4) for tissue slices. Tumoroid scale bar = 50 µm; Tissue scale bar = 100 µm. **E** Percentage of cells expressing the different markers in tumoroids and tissue for the human tumor NJ115. Quantification of different populations was made by the Thermo Fisher’s HCS studio software using the spot detector bioapplication. Reference levels for positive cells to each marker was the same for all samples. Quantification was made on the following number of replicates: Tumoroids = 6–8; Tissues = 2. Statistical analysis was made using student *t*-test, *p < *0.05, **p < 0.01, ***p < 0.001, ****p < 0.0001.

Using our scaffold-free tumoroid method, multiple patient-derived renal cancer tumoroids were successfully generated with high efficiency (90%) from patient’s tumor samples (Fig. S2A). Among them, NJ115 was identified as a grade pT1a ccRCC by pathologists. H&E staining revealed that the renal clear cell phenotype of the original NJ115 tissue was conserved in the tumoroids (Fig. 7A). NJ115 tumoroids were sensitive to Sunitinib only (Fig.7B) while corresponding organotypic slice culture was sensitive to both KU-CX and Sunitinib (Fig. 7C), unveiling differences in drug sensitivity of both models. We performed immunofluorescence labeling on NJ115 tumoroids and the original tumor tissue using different biomarkers (Fig. 7D). Surprisingly, grouped analysis of their expression levels in NJ115 tumoroids compared to the original tumor tissue (Fig. 7E) showed a decrease in E-cadherin expression, lack of CD44^+^ progenitor-like cells and absence of cell proliferation. In contrast, we found similar expression levels of FAP, Vimentin, CAIX, CD68, CD31 and Pan Collagen.

In MZ076 and CM095 tumoroids, intratumor CD31^+^ cells were present. Moreover, infiltrating CD8^+^ cells were detected in MZ076 tumoroids (Fig. S2B). Collectively, this means that multiple cell types and matrix components such as fibroblasts, cancer cells, macrophages, T-lymphocytes, endothelial cells and de novo collagen production were also maintained in patient-derived tumoroids. Interestingly, the addition of F12 and EGF in the culture media seemed sufficient to preserve CD31^+^ endothelial progenitor cells that may participate to the formation of endothelial venules (see Fig. 7E and S2B).

Altogether, these data indicate that it is feasible to produce patient-derived renal cancer tumoroids with high efficiency, even from low-grade tumors. They provide a biologically relevant model system that recapitulates tumor intrinsic characteristics, preserving the cell diversity of the parental tumor. Consequently, they could be used as avatars to screen new drugs.

## Discussion

Evidences were provided that tumoroids derived from primary cell lines lose inter-tumor heterogeneity as well as the appropriate features of the tumor from which they originated [51]. In contrast, the use of functional assays involving personalized ex vivo tumor models such as tumoroids generated from patient tumor samples have shown many advantages to recapitulate patient’s tumors properties as well as to predict patient’s treatment response [21, 52]. They have been proposed as avatars to capture individual patient responses to therapy and for their accuracy to recapitulate cancer heterogeneity [14]. Moreover, they take into account the wider tumor microenvironment, that can modulate treatment response to chemotherapy or even immunotherapy [53].

Here, we demonstrated, for the first time, the feasibility of culturing mice as well as human ccRCC tumoroids, directly from fresh tissue using a scaffold-free method. We could establish a stable culture method using magnets that are known not to be toxic and not to alter treatment response [33, 54]. Moreover, our model preserves the cellular heterogeneity and tumor microenvironment. RNA sequencing showed the reproducibility of the method, highlighting that the transcriptome was well conserved in tumoroids compared to their matching tumor tissue. Patient-derived tumoroids have a weak proliferation index, which is often the case with ccRCC tumors that have a Ki67 positive median of 5% [55]. Importantly, immunofluorescence analysis revealed that they were also able to maintain multiple tumor-resident cell types, including endothelial progenitor cells, which were never reported in other ccRCC tumoroid culture models (Fig. 4B, D). Vasculature establishment usually requires shear stress and microfluidic devices as it was demonstrated for the development of perfused healthy kidney organoids [56]. Our tumoroid model disclosed rare endothelial progenitor cells that were able to form plexus-like structures, suggesting that the tumoroid composition can be further optimized by altering the media conditions. As already reported [57], we found that addition of EGF to patient-derived tumoroids was sufficient to maintain a significant population of endothelial progenitor cells. Lastly, we showed that spheroids generated from different renal cancer cell lines or mice tumoroids provide opportunities to evaluate the invasiveness of renal cancer cells in a 3D physiological context. This analysis revealed that spheroids as well as tumoroids derived from different cell lines did not exhibit the same invasive capacities.

In summary, although tumoroids lack active vascular networks limiting their utility for in-depth study of the effects of immunotherapies, our scaffold-free tumoroid model provides a robust preclinical tool for drug screening and biomarker assessment in line with alternative ex vivo methods like tumor tissue slice culture or in vivo xenograft models. This is timely relevant since Food and Drug Administration no longer has to require animal testing for new drugs to receive approval, supporting alternative methods such as organoids in predicting cancer drug efficacies [58].

## Material and methods

### Cell culture

ccRCC cell lines 786-O, Caki-1 and ACHN were obtained from ATCC (CRL-1932, HTB-46 and CRL-1611 respectively). The cell lines were grown in 10 cm diameter plates in a humidified incubator (37 °C, 5% CO_2_) with RPMI 1640 medium (Gibco) containing 10% of fetal bovine calf serum, penicillin (100 U/mL) and streptomycin (100 µg/mL). RPTEC cell line was obtain from Evercyte and grown in ProXup media (Evercyte). To produce spheroids, cells were counted and coated with magnetic NanoShuttle (Greiner) (1 µL per 20,000 cells) thanks to 3 centrifugation/resuspension cycles at 400 g for 5 min each. Cells were seeded into U-bottom 96-well plates coated with 20 mg/mL poly(2-hydroxyethyl methacrylate) (PolyHema) (Sigma-Aldrich) at the density of 1500 cells per well, centrifuged at 400 g for 5 min and then were grown for 3 days.

### Generation of luciferase expressing cells

786-O and ACHN cells were plated into 6-well plates in 2 mL of serum-supplemented RPMI 1640 medium. The day after, adherent cells were infected with Lenti-II-CMV-Luc-IRES-GFP virus (ABM, Deutschland) (1–5 MOI (multiplicity of infection)) diluted in 1 mL of serum-supplemented medium containing 8 µg/mL of polybrene (Sigma). After 4 h, 1 mL of medium was added to each well and transduction was maintained for 16 h before changing the medium. For stable transduction, puromycin selection started 36 h post-infection (at the concentration of 2 µg/mL) and maintained for 2 weeks.

### Mice orthotopic and subcutaneous tumor xenograft model

All animal studies were approved by the institutional guidelines and those formulated by the European Community for the Use of Experimental Animals. Renal orthotopic implantation was carried out by injection of 1.10^6^ 786-O luc or ACHN luc cells into the left kidney of athymic nude mice (6 weeks-old female BALB/c Nude, CByJ.Cg-Foxn1nu 522 /J; Charles River) as previously described [10, 59]. Subcutaneous xenografts were obtained by injecting 8.10^6^ 786-O luc cells on the back of athymic nude mice. Tumor growth was followed by intraperitoneal injection of 150 mg/kg D-luciferin Potassium salt using IVIS Imager (Perkin Elmer).

### Patients and clinical samples

All human renal carcinoma samples were obtained from patients, with their informed consent, and all procedures were approved by the ethics committee (Patient Protection Committee No. 2017 A0070251). Patients were recruited under the clinical trial, Comborein (NCT03572438) [60]. Fresh renal tumor tissue was obtained from patients undergoing a partial or a total nephrectomy for cancer at the Urology Department, University Hospital Center of Grenoble, Alpes (CHUGA).

### Fresh tissue sectioning

A Vibratome VT1200 (Leica Microsystems) was used to cut thin (300 µm) slices from fresh tumor tissue as previously described [10, 11]. Briefly, samples were soaked in ice-cold sterile-balanced salt solution (HBSS), orientated, mounted and immobilized using cyanoacrylate glue. Slicing speed was comprised between 0.03–0.08 mm/s depending on tissue stiffness. Vibration amplitude was set between 2.95 and 3.0 mm.

### Organotypic tissue cultures

Tissue slices were cultured as previously described [10, 11]. Briefly, they were cultured on organotypic inserts for 48 h (one slice per insert; Millipore) at 37 °C in a 5% CO_2_ humidified incubator under agitation, using 2 mL of DMEM media supplemented with 10% inactivated fetal bovine serum (FBS) (GIBCO), 100 U/mL penicillin + streptomycin (Invitrogen). The slices were incubated with the following compounds dissolved in DMSO: KU-60019 (10 µM; Selleck Chem) plus CX-4945 (10 µM; Plateau Synthèse Organique, UGA); Sunitinib (10 µM; Selleck Chem) and DMSO (0.1%; Sigma) as control. After 48 h the viability of tumor slices was assayed [11].

### Cell dissociation and tumoroids production

Cell dissociation was perform thanks to the gentleMACS Dissociator and the Tumor dissociation kit (Miltenyl Biotec) according to manufacturer’s instructions. Briefly, pieces of fresh tumors from mice or patients were chopped with a scalpel and transferred into the “C” dissociation tube. Chopped tumor was suspended in supplemented DMEM and enzymes from the kit (A, H and R). Mechanical dissociation was performed by 3 cycles of stirring using the GentleMACS Dissociator. Between each cycle, the tube was incubated 30 min at 37 °C for enzymatic digestion. Cell suspension was then filtered and washed through the MACS SmartStrainers (70 µm pores) to remove undissociated particles. Cells were centrifuged at 300 g for 5 min and Red Blood Cells (RBC) were lysed thanks to the RBC Lysis buffer during 4 min at 4 °C. The reaction was stopped by PBS addition. Cells were counted and coated with magnetic NanoShuttle (Greiner) (1 µL per 20,000 cells) thanks to 3 centrifugation/ resuspension cycles at 400 g for 5 min each. Cells were seeded into flat repellent 96-well plates (Greiner) at the density of 10,000 cells per well. For tumoroids formation, plates were maintained on the Spheroid Drive support (Greiner) for 14 days at 37 °C with 5% CO_2_ in a humidified incubator as described in [33]. Mice tumoroids were cultured in DMEM media supplemented with 10% FBS (GIBCO), 100 U/mL penicillin + streptomycin (Invitrogen), while human tumoroids were cultured in DMEM/F12 (1/1) supplemented with 10% FBS (GIBCO), 100 U/mL penicillin + streptomycin, and 10 ng/mL EGF.

### Three-dimension (3D) cell death measurements

Three days-old spheroids (786-O and ACHN) and 14 days-old tumoroids were transferred into a U-bottom 96-well plate coated with 20 mg/mL polyHema. They were treated for 48 h, with KU-60019 (10 µM) plus CX-4945 (10 µM) or Sunitinib (10 µM) or DMSO (0.1%) as control. To assess cell death, 0.5 µg/mL Propidium iodide (PI) was added to the culture during all the treatment. PI incorporation was followed in real time with the Incucyte ZOOM® imager.

### Invasion assay

Three days-old spheroids and 14 days-old tumoroids were included in TissueSpec Kidney^®^ hydrogel (Xylyx Bio, New York, USA). At day 0 and 7, cells were stained with Live and Dead viability kit containing Calcein and Ethidium Homodimer (Invitrogen). They were also stained with CMAC t-BOC-Leu-Met (Invitrogen) for calpain activity measurement. CMAC is a calpain peptidase substrate that will fluoresce when cleaved by active calpain [46]. Image analysis was performed using Icy and ImageJ softwares.

### Cryomicrotome sectioning

Pieces of fresh tumors and tumoroids were fixed in paraformaldehyde 4% (Sigma) for 1 h at room temperature. The pieces were included in OCT mounting medium and frozen in liquid nitrogen up to solidification. Tissues and tumoroids were conserved at −80 °C before use. Slices (8 µm thick) were cut with a Cryo-microtome (Leica) to perform immunofluorescence and Hematoxylin and Eosin staining for tumor characterization.

### Immunofluorescence labeling of tumoroids

Tumoroids were labeled one by one in a flat repellent 96-well plate. For all liquid removing steps, we used the Handling Magnetic support (Greiner) to keep tumoroids inside the wells while removing the solutions. Tumoroids were washed in PBS and fixed in paraformaldehyde 4% (Sigma) for 1 h at 4 °C. All the next steps were performed under horizontal agitation (60 rpm). After 10 min in PBS-Tween20 0.1%, they were permeabilized and saturated 1 h in Organoid Washing Buffer (OWB) containing 0.1% Triton-X100 and 0.2% BSA diluted in PBS. Primary antibodies were incubated overnight at 4 °C. On the second day, tumoroids were washed 3 times during 2 h in OWB and secondary antibodies were incubated overnight at 4 °C. On the third day, tumoroids were washed 3 times and during the second one, Hoechst was added to OWB (1/1000^e^) for nuclei staining. At the end, tumoroids were transferred into 96-CellView Microplate glass bottom (Greiner) for fluorescence imaging. The primary antibodies used are the following ones : FAP (Abcam #ab53006, 1/100^e^), Vimentin (Sigma #V5255, 1/200^e^), CD31 (Abcam #ab28364, 1/25^e^), E-cadherin (BD #610181, 1/200^e^), PanCollagen (ThermoFischer #PA5104252, 1/100^e^), CD44 (SantaCruz #sc-53298, 1/200^e^), CA9 (NovusBio #NB100-417, 1/500^e^), Ki67 (DAKO #M7240, 1/100^e^), CD8 (Abcam #ab101500, 1/100^e^), CD68 (ThermoFisher #PA5-32330, 1/100^e^) and Phalloidin (Invitrogen #A12379, 1/400^e^). Secondary antibodies are the following ones: Cy3 Goat anti-rabbit IgG (Jackson ImmunoResearch #111-165-003, 1/1000^e^), 488 Goat anti-mouse IgG (Invitrogen #a11001, 1/2000^e^) and 488 Goat anti-Mouse IgM µ chain (Invitrogen #a21042, 1/2000^e^).

### Immunofluorescence labeling of cryomicrotome slices

Frozen slices were allowed to stabilize at room temperature (RT) for 10 min. They were washed 3 times with PBS (1X) and then permeabilized in PBS Triton-X100 0.5% for 10 min at RT. After 3 washes in PBS-Tween20 0.05%, they were saturated with PBS-Tween20 0.05%, 5% SVF and 0.2% BSA for 1 h at RT. Antibodies were incubated overnight at 4 °C. The day after, slices were washed 3 times in PBS-Tween20 0.05% and incubated for 1 h at RT in the dark with secondary antibodies. Slices were washed 5 times in PBS-Tween20 0.05% and nuclei staining was performed with Hoechst for 15 min at RT in the dark. After a final wash, slices were mounted for microscopy using Fluoromount-G (Southern Biotech). Antibodies used were the same as described for tumoroids labeling.

### Hematoxylin and Eosin staining

Frozen slices were allowed to stabilize at room temperature (RT) for 10 min and washed in water to remove OCT mounting medium. Slices were stain for 40 s with Hematoxylin (Sigma) followed by washing using water and stain for 4 min using Eosin (Sigma). Slices were then dehydrated in ethanol 100% for 2 × 5 min and with Xylene for 2 × 2 min. Slices were mounted using Merkoglass (Sigma) and visualized under microscope.

### Fluorescence microscopy

Qualitative imaging was performed thanks to the confocal microscope LSM 880 with AiryScan module (Zeiss) at the µLife platform, CEA Grenoble. Pictures were taken using the 40X oil objective. Quantitative imaging was performed thanks to the CX7 Cell Insight high throughput confocal microscope (ThermoFisher) at the CMBA platform, CEA Grenoble. Pictures were taken using the 20X air objective (NA 0.45) for tumoroids and 10X air objective (NA 0.4) for tissue slices. Image quantification was performed with the Thermo Scientific HCS studio Cellomics Software by using the spot detector bio-application.

### RNA extraction

RNA extracts from tumoroids were obtained using the RNeasy Micro Kit (Qiagen) following manufacturer’s instructions. Two tumoroids were used for each extract. RNA Extracts from tissues were obtained using the MirVana PARIS kit (ThermoFisher).

### Bulk RNA Barcoding (BRB)-sequencing and raw data preprocessing

BRB-sequencing was perfomed at the Research Institute for Environmental and Occupational Health (Irset, Rennes, France) as previously described [10, 61]. The first read contains 16 bases that must have a quality score >10. The first 6 bp correspond to a unique sample-specific barcode and the following 10 bp to a unique molecular identifier (UMI). The second reads were aligned to the human reference transcriptome from the UCSC website (release hg38) using BWA version 0.7.4.4 with the non-default parameter “−l 24”. The second reads were also aligned to the mouse reference transcriptome from the UCSC website (release mm10) using BWA version 0.7.4.4 with the non-default parameter “−l 24”. Reads mapping to several positions in the genome were filtered out from the analysis. The pipeline is described in Reference [10.1016/j.chemosphere.2020.128468]. After quality control and data preprocessing, a gene count matrix was generated by counting the number of unique UMIs associated with each gene (lines) for each sample (columns). The resulting UMI matrix was further normalized by using the rlog transformation implemented in the DeSeq2 package [10.1186/s13059-014-0550-8]. Raw and preprocessed data were deposited at the ArrayExpress repository under the accession number E-MTAB-12928.

### Bioinformatics analysis

The hcut method of the factoextra package on R (version R 4. 1) with 2-3 clusters is implemented on the original tumor tissue samples (A1M, A2M and 71 M) and on the tumoroids samples (A1T1, A1T2, A1T3, A1T4, A2T1, A2T2, A2T3, A2T4, 71T1, 71T2, 71T3), for the human genome (11443 genes) and for the mouse genome (11914 genes) independently. This method computes hierarchical clustering and splits the tree into 3 clusters. It also accepts distance measurement methods based on pearson correlation. To visualize the results of the hierarchical clustering, we use the fviz_dend method of the R package factoextra (version R 4.1). This method allows creating dendograms. We have chosen to perform the hierarchical clustering and the dendrogram with three clusters. Indeed, a preliminary analysis of the shape of the dendrogram gave us an indication on the number of clusters to retain and then we calculated the inertia of the clustering tree. The result of these two observations was to realize the hierarchical clustering with three clusters. The distinct intersections of set of genes or pathways were visualized by the make_comb_mat() function with a mode parameter set to “distinct” followed by the UpSet() function from the ComplexHeatmap R/Bioconductor package (version 2.14.0). Intersection plots were generated using the UpsetPlot R package.

### Statistical analysis

The statistical significance of differences between the means of two groups was evaluated using GraphPad version 9. Tests are indicated below in each figure legend.

### Supplementary information

Supplementary Figure 1

Supplementary Figure 2

Supplementary Table 1

Supplementary Table 2

## Data Availability

All data generated or analyzed during this study are included in this published article or are available at the ArrayExpress repository under the accession number E-MTAB-12928.
