# Supplementary figures and images for: A new scaffold-free tumoroid model provides a robust preclinical tool to investigate invasion and drug response in Renal Cell Carcinoma
Source: Cell Death Dis. 2023 Sep 22;14(9):622. doi: 10.1038/s41419-023-06133-z (PMC10517165; doi:10.1038/s41419-023-06133-z)

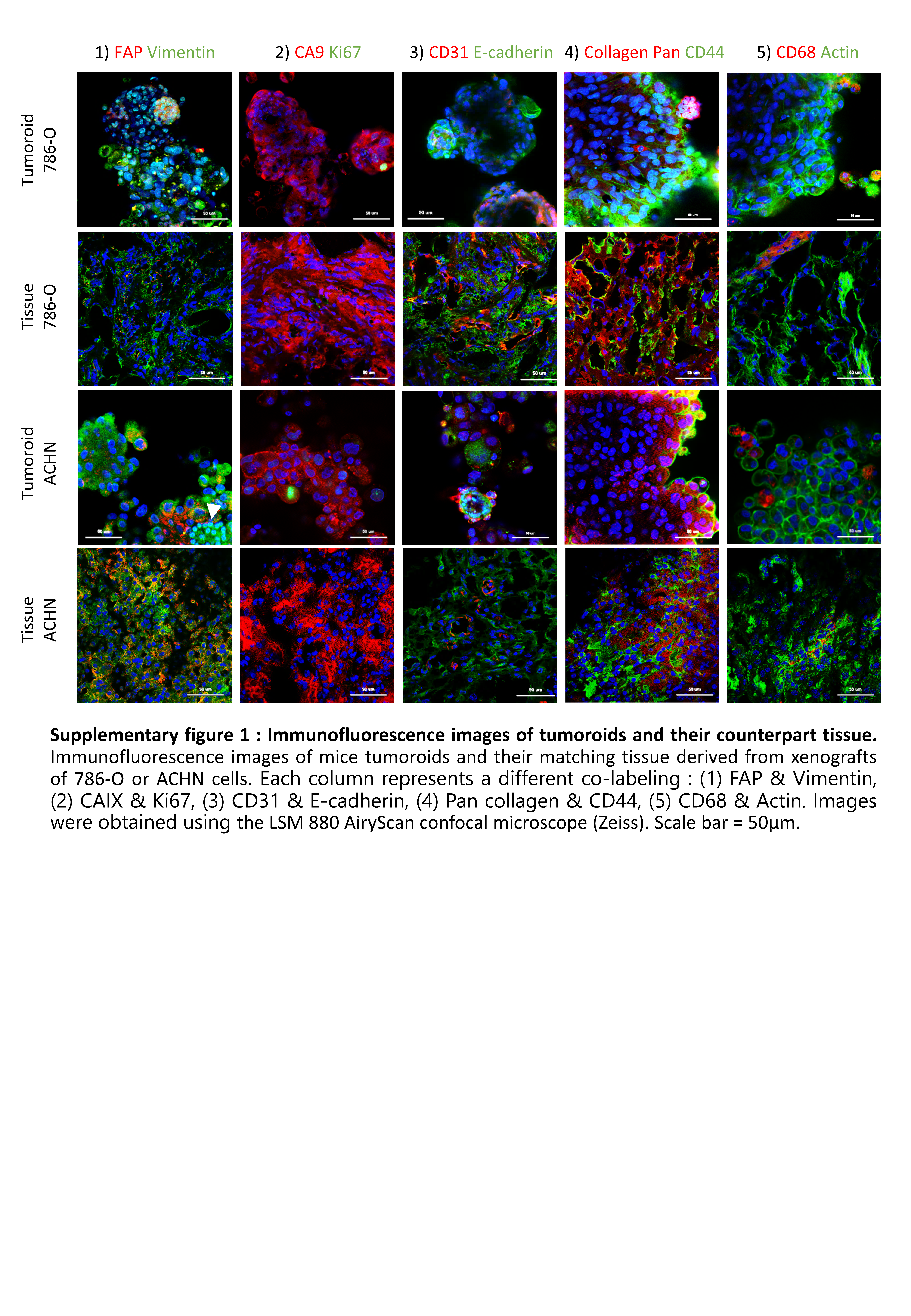

Supplement: Supplementary file 1 — Supplementary Figure 1 [file 41419_2023_6133_MOESM1_ESM.tif]

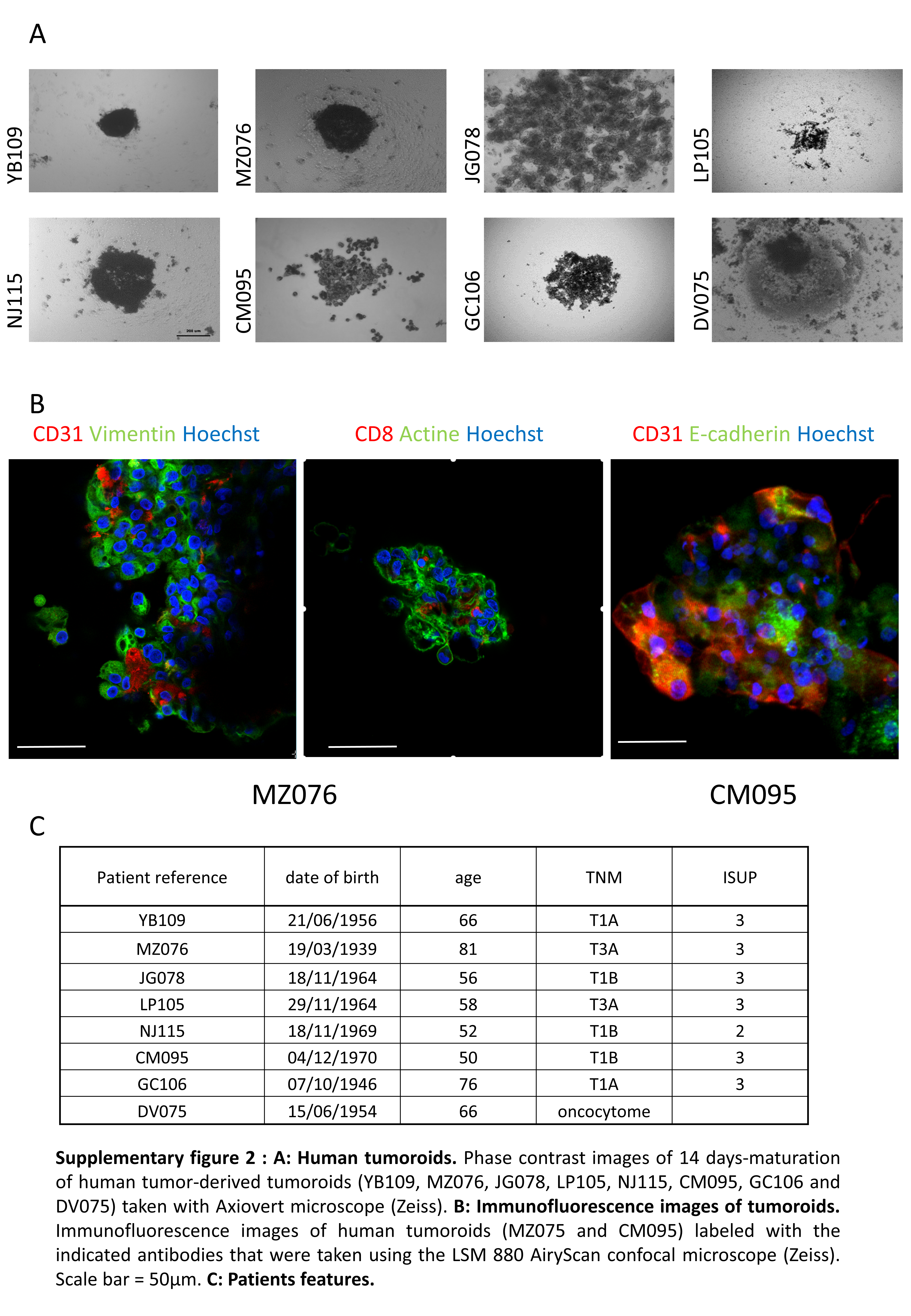

Supplement: Supplementary file 2 — Supplementary Figure 2 [file 41419_2023_6133_MOESM2_ESM.tif]
